# Supplementary material for: Unusual Partners: γδ-TCR-Based T Cell Therapy in Combination with Oncolytic Virus Treatment for Diffuse Midline Gliomas
Source: Int J Mol Sci. 2025 Feb 28;26(5):2167. doi: 10.3390/ijms26052167 (PMC11900589; doi:10.3390/ijms26052167)
Supplement: Supplementary file 1 [file ijms-26-02167-s001.zip › ijms-3407226-supplementary.pdf]

Supplementary data S1

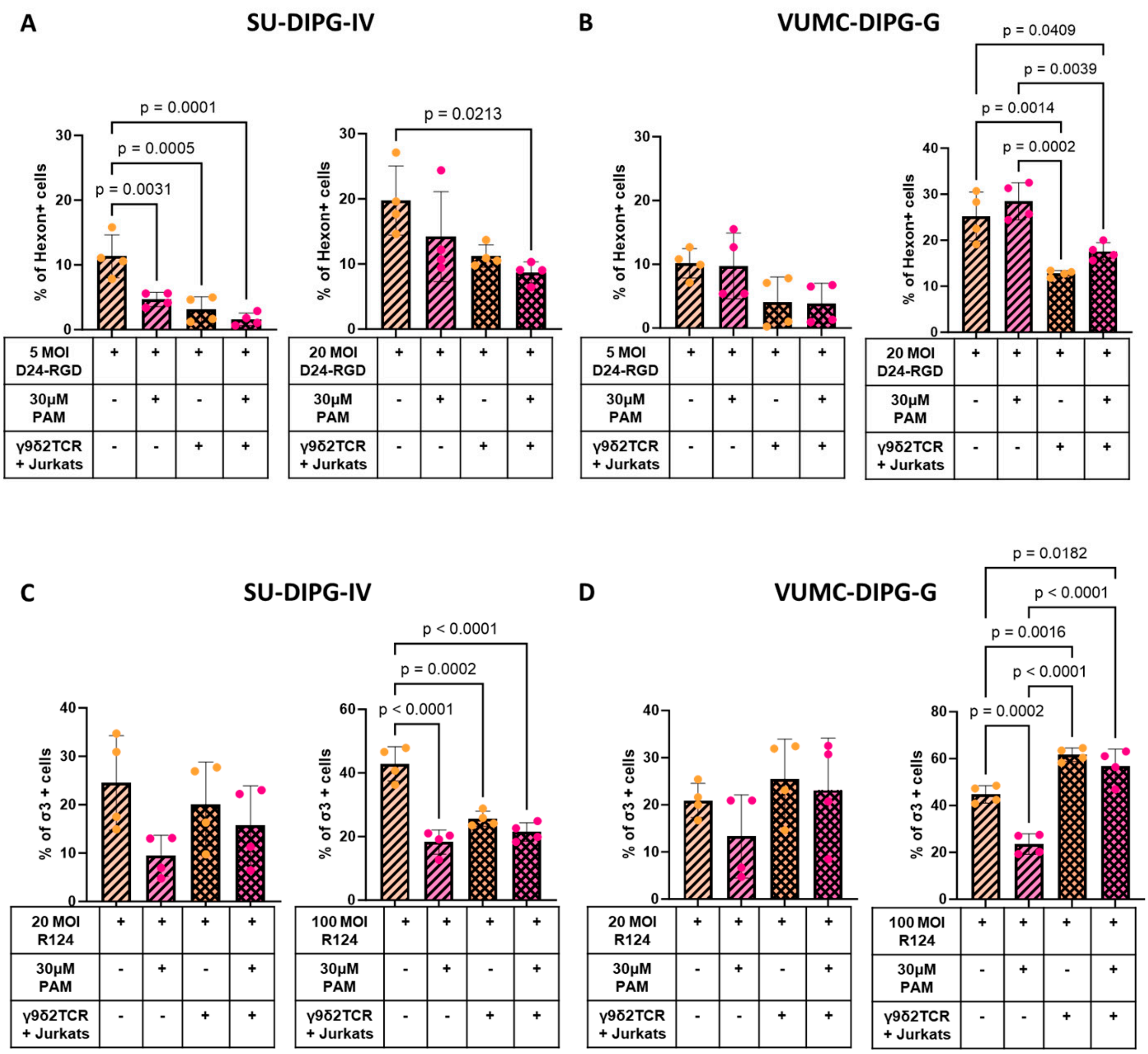

Supplementary Figure S1: Reduced D24-RGD and R124 infection rate in the presence of 30μM PAM and Jurkats expressing γ9δ2 TCR. Percentages of Hexon+ SU-DIPG-IV (A) and VUMC-DIPG-G (B) respectively, infected with 5 MOI D24-RGD or 20 MOI D24-RGD for 48h in conditions including no PAM, no PAM with 1:1 ratio DMG:Jurkats, 30μM PAM and 30μM PAM with 1:1 ratio DMG:Jurkats. Percentages of σ3+ SU-DIPG-IV (C) and VUMC-DIPG-G (D) respectively, infected with 20 MOI R124 or 100 MOI R124 for 48h in conditions including no PAM, no PAM with 1:1 ratio DMG:Jurkat cells, 30μM PAM and 30μM PAM with 1:1 ratio DMG:Jurkat cells. The experiments are represented as dot points (biological replicates) and error bar as ± SD from n = 2 independent experiments and statistics were assed with one-way ANOVA and corrected for multiple comparisons with Tukey test.

Supplementary data S2

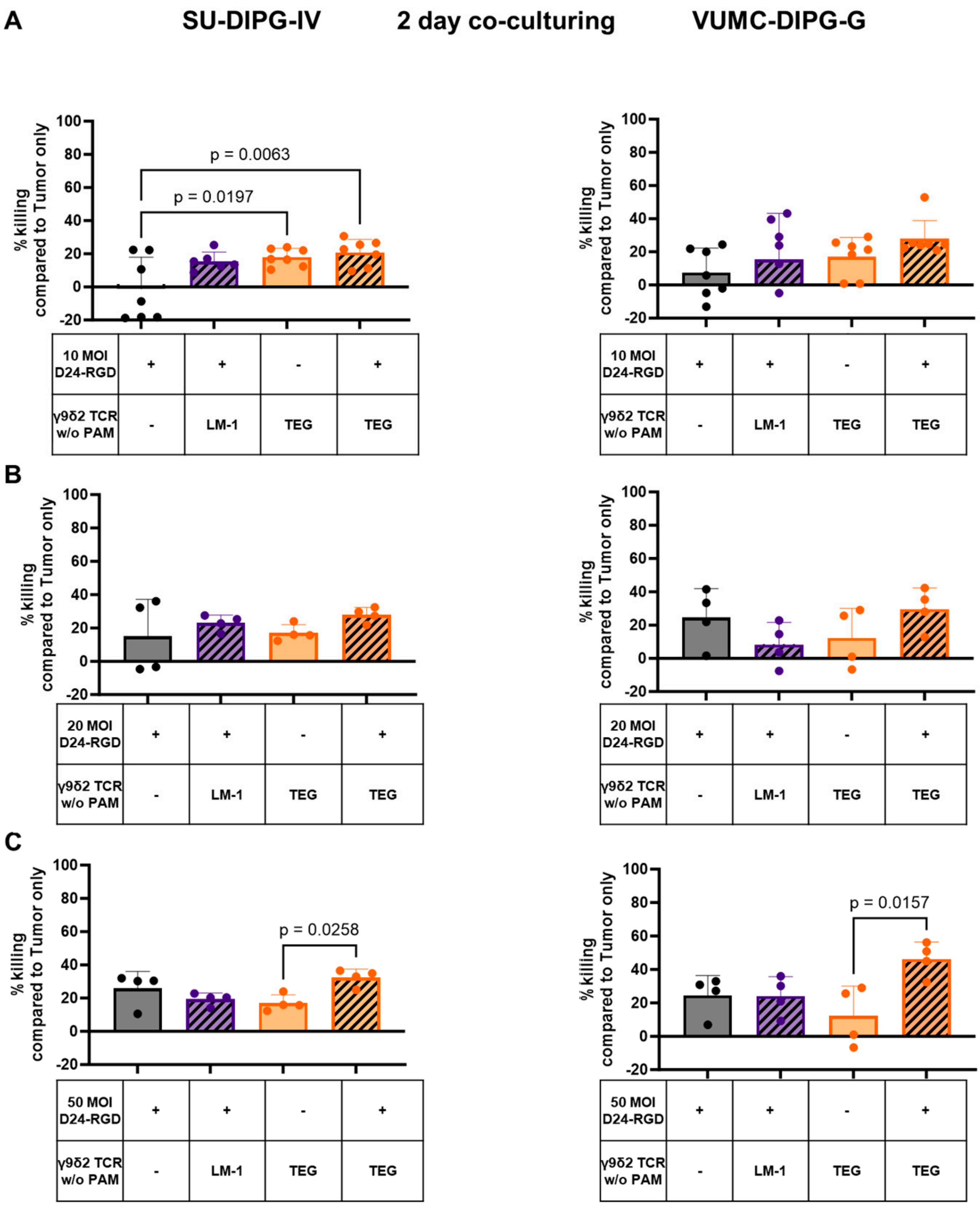

Supplementary Figure S2: Improved killing benefit of combining TEGs with D24-RGD is lost in higher concentrations of D24-RGD. Killing assays comparing 10 MOI (A), 20 MOI (B), 50 MOI (C) D24-RGD monotherapy, TEG monotherapy, combination of D24-RGD with LM-1 and TEG cells. Data for killing assays from multiple independent experiments (n=3) for SU-DIPG-IV and VUMC-DIPG-G for figures A while (n=2) for figures B&C represented as dot points (biological replicates) and error bar as ± SD. Statistics were assessed with the one-way ANOVA and corrected for multiple comparisons with the Tukey test.

Supplementary data S3

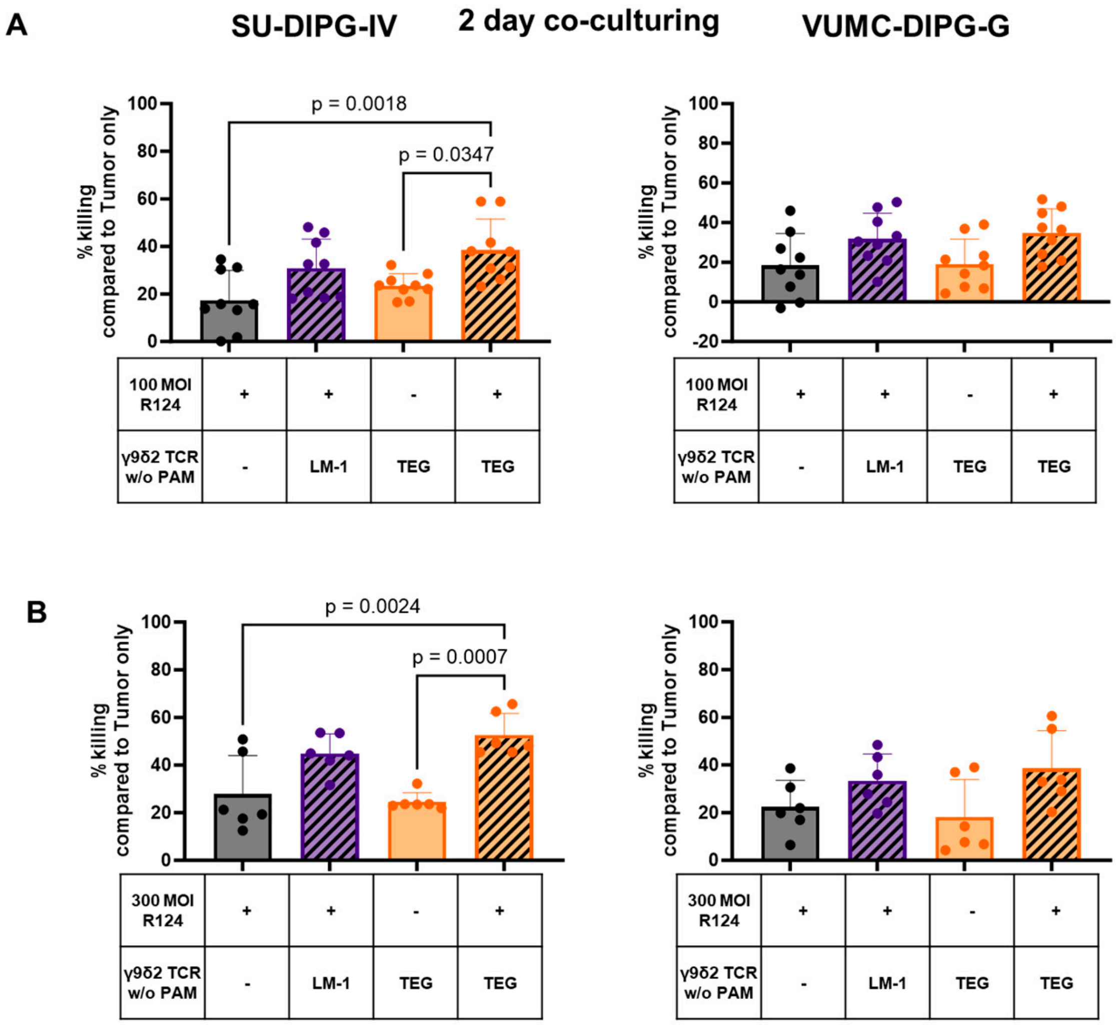

Supplementary Figure S3: Improved killing benefit of combining TEGs with R124 is lost in higher concentrations of R124. Killing assays comparing 100 MOI (A), 200 MOI (B) R124 monotherapy, TEG monotherapy, combination of R124 with LM-1 and TEG cells Data for killing assays from multiple independent experiments (n=4) for SU-DIPG-IV and VUMC-DIPG-G for figures A while (n=3) for figures B represented as dot points (biological replicates) and error bar as ± SD. Statistics were assessed with the one-way ANOVA and corrected for multiple comparisons with the Tukey test.

Infection Rate Assay

DMG cells were collected, washed in cold PBS, and dissociated into single cells with accutase for 5 min at 37 °C and 5% CO_2, and then stained with 5 μM of CTV for 20 min at 37 °C and 5% CO_2. The cells were counted using trypan blue 0.4%, and 25.000 tumor cells/well were seeded in 96 wells in TSM. After 1 h incubation, the DMG cells were infected with 5/20 MOI of D24-RGD or 20/100 MOI R124 or 30 μM PAM or 25.000 Jurkat-76-γδTCR cells for the respective conditions for 48 h. At 48 h post-infection, the DMG cells were centrifuged, the supernatant was removed, and the cell pellet was washed in cold PBS and treated with accutase for 5 min at 37 °C and 5% CO_2. After dissociation, the cells were washed with PBS and stained with 7-AAD-PE-Cy5 for 30 at 4 °C. After staining, cells were washed with PBS and fixed in 1% PFA for at least 20 mins at 4 °C. The fixed cells were subsequently washed and incubated in permeabilization buffer (5%FBS, 1% Triton-X in PBS) for 20 minutes at 4 °C. Then, cells were washed with permeabilization buffer and stained for D24-RGD with anti-Hexon Adenovirus type 5 (AB1056, Sigma-Aldrich, Amsterdam, The Netherlands) in permeabilization buffer, and for R124 with anti-σ3 T3D reovirus antibody (4F2, DSHB, Iowa City, IA, USA) in permeabilization buffer for 30 minutes at 4 °C. After washing, cells were stained with secondary antibodies conjugated with AF-488, anti-goat Igg for Hexon (11055, Thermo Fisher, Utrecht, The Netherlands) or anti-mouse Igg for σ3 (11001, Thermo Fisher, Utrecht, The Netherlands) respectively. Finally, the plates containing the fixated cells were analyzed with flow cytometry using the Cytoflex; FACs gating allowed the measurement of Hexon+ and σ3+ % of D24-RGD and R124 infected DMG respectively (CTV+ & 7-AAD- cells), while excluding the dead cells, analyses was processed in FlowJo.
